# 

**Published:** 1885-03

**Authors:** 


10 cts. a copy. **MARCH, 1885.** $1.00 per Year.

### HALL'S JOURNAL of HEALTH

ESTABLISHED 1854

Office, 75 & 77 Barclay Street. New York.

